# Fin whale song characteristics and potential subpopulation identity in the New York Bight

**DOI:** 10.1038/s41598-024-52228-8

**Published:** 2024-02-13

**Authors:** Carissa D. King-Nolan, Melinda L. Rekdahl, Anita Murray, Samantha Strindberg, Mark F. Baumgartner, Howard C. Rosenbaum

**Affiliations:** 1 Ocean Giants Program, Wildlife Conservation Society, Bronx, NY 10460 USA; 2 Biology Department, Woods Hole Oceanographic Institution, Woods Hole, MA 02543 USA; 3 Department of Ecology, Evolution and Environmental Biology, Columbia University, New York, NY 10027 USA; 4 Present Address: Marine Mammal Research, Maine Department of Marine Resources, West Boothbay Harbor, ME 04575 USA

**Keywords:** Behavioural ecology, Marine mammals, Marine biology

## Abstract

Fin whale (*Balaenoptera physalus*) song can follow a highly consistent pattern, and regional differences in song patterns can be a valuable indicator of subpopulation identity and distribution. In the Northwest Atlantic, endangered fin whales are currently managed as a single stock despite previous identification of different regional song patterns, which indicates potential subpopulation structuring and vulnerability to anthropogenic disturbance if not managed accordingly. Here we document fin whale song in the New York Bight (NYB) from 2017 to 2020 using passive acoustic data to identify monthly and yearly trends in song patterns and to explore potential subpopulation structuring. The predominant song pattern observed was highly consistent with the pattern documented almost a decade prior in the NYB, with short inter-note intervals (INI) from fall–winter and long-INIs in the spring. However, in one song year the majority of songs were composed of long-INIs. This change in song pattern could be due to a shift in fin whale behavior or possibly multiple fin whale subpopulations using the NYB. Fin whales in the NYB may be particularly vulnerable to disturbance given the increasing anthropogenic pressures in this region, and further research into subpopulation structuring is needed to ensure adequate management of these endangered whales.

## Introduction

Marine ecosystems around the world are heavily impacted by anthropogenic activities, particularly coastal areas^1^. These activities include commercial shipping, recreational and commercial fisheries, maritime industrial development (e.g., offshore wind development), and coastal development^1,2^. As anthropogenic use of marine ecosystems increases, there is a need to develop and implement effective management strategies to mitigate potential impacts on marine species^2^. However, effective management strategies require knowledge of the distribution of species, particularly of biologically meaningful populations that may be more vulnerable to local disturbance^3–7^.

Fin whales (*Balaenoptera physalus*) have a global pelagic distribution^3,8^, and are currently listed as endangered under the U.S. Endangered Species Act^9^. Fin whales sing a highly stereotyped song that is seasonally a major component of the ocean soundscape^10–12^. As such, passive acoustic monitoring (PAM) is a useful method for examining fin whale presence and distribution^13–15^. Only male fin whales have been documented singing and song is believed to serve a reproductive function^6,8,13,16–19^. Assessments of fin whale song have documented regional differences, and these differences have been used to distinguish acoustic groups or subpopulations^3,6,20^ (hereafter referred to as subpopulations) both across ocean basins and between adjacent geographic areas^21–25^. Furthermore, these differences in song can reflect more fine-scale temporal changes in social behavior and/or movement among populations at time scales that are shorter (i.e., decades) than those required to show genetic change or divergence^3,4^.

Fin whale song is composed of downsweeps generally ranging from 23 to 18 Hz, and with a center frequency around 20 Hz these downsweeps are generally referred to as 20 Hz pulses or notes^8,19,21,25,26^. Song sequences are composed of long, repeated series of 20 Hz notes, and within a song sequence brief periods of silence (lasting from seconds to minutes) can occur when the whale surfaces to breathe^6,8,16,23^. Different song sequences are separated by much longer periods of silence that typically last for several hours^6,20^. Long song sequences are generally recorded leading up to and during the winter reproductive season while shorter, more sporadic song sequences are recorded during the summer^8,13,16,23,26^. Song is typically characterized by the inter-note interval (INI), or length of time, between two sequential 20 Hz notes^3,6,19,23,25^. The general patterning of INIs within a song sequence can be described in several ways including as a singlet pattern (consistent INIs), a doublet pattern (alternating INIs), or more simply as INI pattern^8,16,20,24,27,28^. Hereafter, INI pattern will be used when referring to the pattern of INIs within song sequences. On larger temporal and/or geographic scales, INI patterns can be more collectively described as song patterns^27^.

Fin whale song in some regions can include seasonal or yearly shifts in INI patterns. Gradual shifts across months within a single year and/or consistent seasonal shifts in INI have been documented in a number of regions^18,19,23,24^. For example, in the Pacific Ocean, Oleson et al*.*^18^ documented a gradual increase in INI from October to March, and INI would reset to approximately the same starting duration each October, which is just prior to the presumed reproductive season. Širović et al*.*^24^ also documented this seasonally shifting song pattern, and they classified it as a distinct song pattern (i.e., long doublet song) compared to the three other song patterns they documented (i.e., short doublet and short/long triplet song). In the northwestern Atlantic Ocean, Morano et al.^23^ similarly observed a song pattern with seasonal shifts in INI with songs characterized by longer INIs from March to May (i.e., spring) and shorter INIs from September to January (i.e., fall through winter). Song patterns that include seasonal shifts in INI may be a reflection of fin whale behavior given that shifts in INI have been hypothesized to be due to changes in behavior and/or reproductive state^17,18,23^. However, not all song patterns include seasonal shifts in INI. Instead, some song patterns exhibit changes in INI between two years and/or across several years^19,24^. Weirathmueller et al*.*^19^ documented a gradual increase in INI and a decrease in the peak frequency of 20 Hz notes across a decade. Likewise, Širović et al.^24^ found a significant increase in INI across six years within the short doublet song pattern they identified. These observed differences in song, both seasonally and yearly, show that some variation in song pattern can be expected, thus an understanding of long-term trends in song pattern is important when using song as an indicator of subpopulation identity and distribution^19,24,28^.

**Figure 1:**
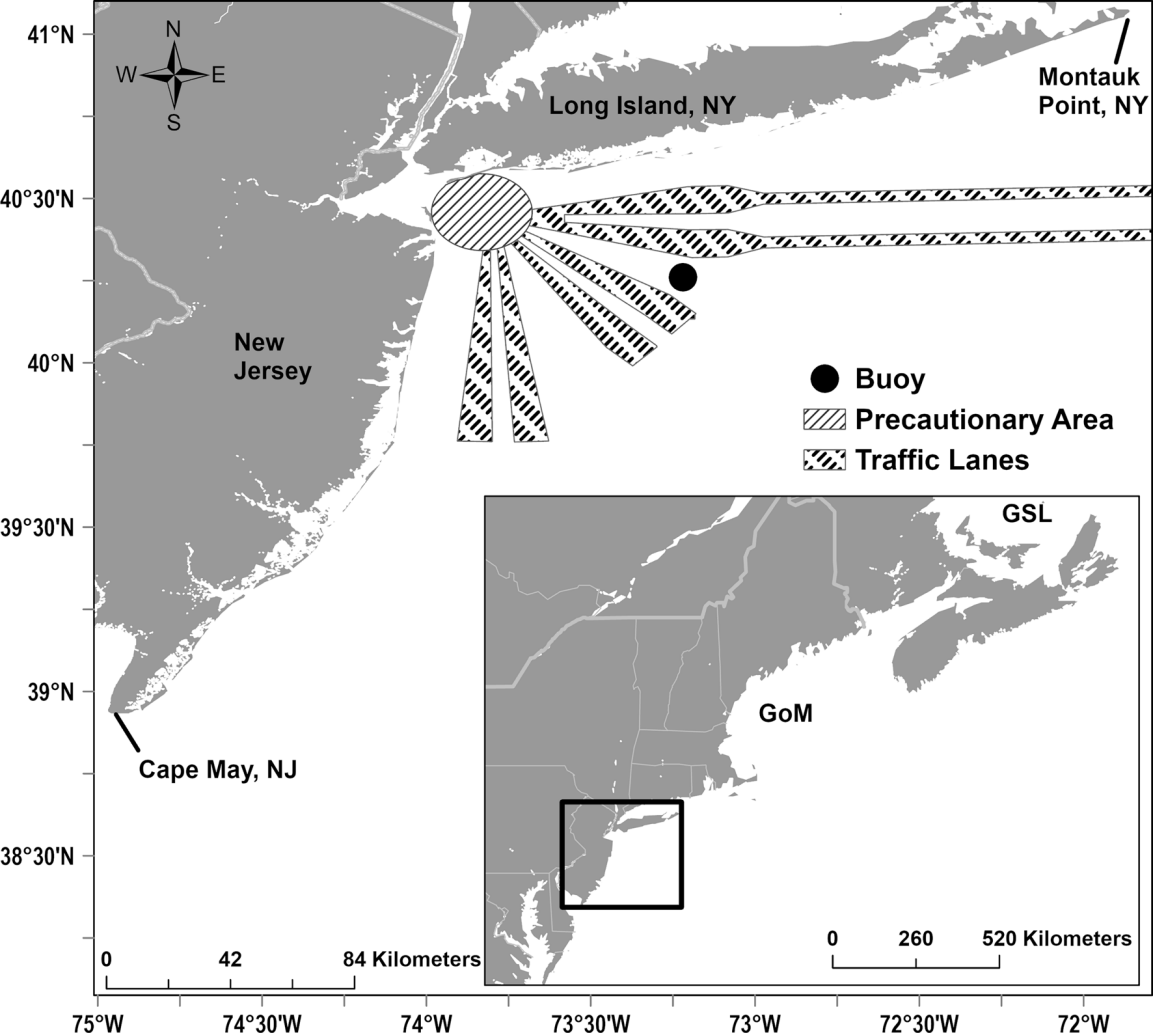
Map of the NYB. The buoy location from January 2017 to December 2020 is shown by the black circle, and nearby vessel precautionary area and traffic lanes shown by the hatched regions leading into the Port of New York-New Jersey. On the inset map, the location of the NYB is shown by the black rectangle, and the locations of the GoM and GSL are labeled. Map generated using ArcGIS Pro (version 3.0.3; https://www.esri.com/en-us/arcgis/products/arcgis-pro/overview).

In the northwestern Atlantic Ocean, the International Whaling Commission (IWC) and the National Oceanic and Atmospheric Administration (NOAA) currently recognize a single fin whale stock^9^ (i.e., the Western North Atlantic stock). However, fin whale song differed between the Gulf of St. Lawrence (GSL) and the Gulf of Maine (GoM) regions^6^, and was similar between the GoM and the New York Bight (NYB) regions^23^. Thus, it is likely that at least two different subpopulations (one within the GSL and one within the GoM/NYB; Fig. 1) of fin whales are present within the northern region of the western North Atlantic Ocean^6,23^, but are managed as a single stock. Managing fin whales in this region as a single stock may leave regional subpopulations of this endangered species vulnerable to local disturbance or extirpation^5^. Investigating long-term trends in song pattern in this region will contribute valuable information on the stability of, or potential changes to, song and will further expand upon the currently limited understanding of subpopulation structure in this region.

Here we investigate the monthly and yearly trends in fin whale song in the NYB from January 2017 to December 2020, and compare these trends with those documented almost a decade prior by Morano et al.^23^ in order to explore long-term trends in song pattern as well as potential subpopulation structuring and distribution in this region. Considering the continued and expanding anthropogenic pressures in the highly urbanized NYB region, such as those associated with forthcoming offshore wind development^29^, there is an urgent need for informed conservation actions, especially for local populations of endangered whales. Investigating the long-term song pattern trends as well as potential subpopulation structuring and distribution of fin whales in this region will support efforts to develop and implement effective management and mitigation practices for this endangered species.

## Results

### Trends in song INI

**Figure 2:**
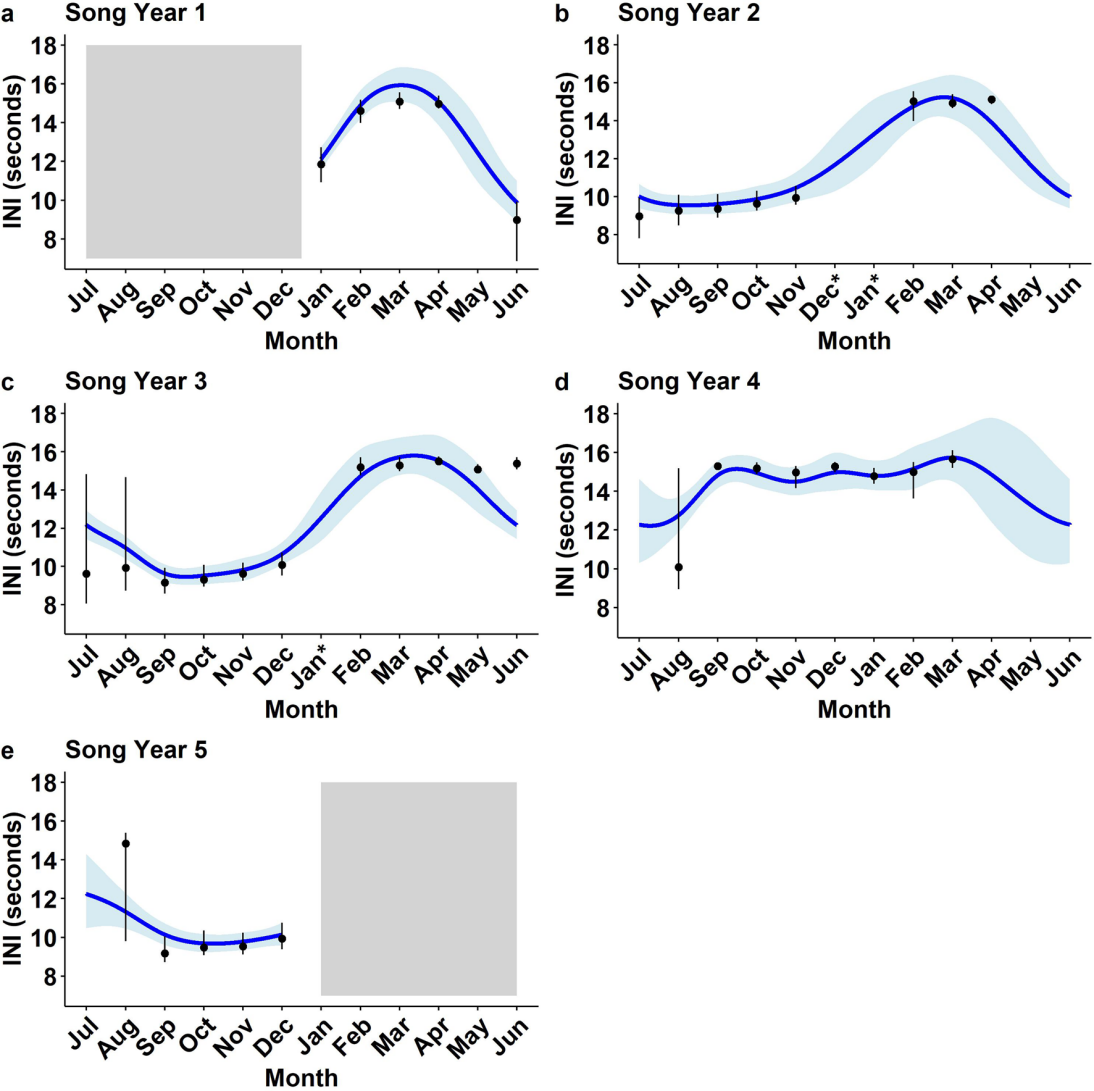
Monthly trends in INI with the expected values predicted by a GAMM for (**a**) song year one (January–June 2017), (**b**) song year two (July 2017–June 2018), (**c**) song year three (July 2018–June 2019), (**d**) song year four (July 2019–June 2020), and (**e**) song year five (July–December 2020). The observed median, 25th quartile, and 75th quartile INIs for each month are shown by the points and whiskers, the blue line is the trend in INI predicted by the best fitting GAMM model, and the light blue shading indicates the 95% confidence interval of the model predicted values. The asterisk on the x-axis indicates months when the buoy was not in the water, with no archival data available from December 2017, January 2018, and January 2019. Song data were not analyzed between July–December 2016 and January–June 2021, indicated by the gray shading.

Archived recordings from a total of 653 days (1959 h) were manually reviewed for the presence of fin whale songs from 2017 to 2020, and 251 annotated fin whale songs were used to assess monthly and yearly trends in song INI (Table 1). Fin whale song was detected in every month of the year, with song most prevalent in the fall to winter (September–December), followed by spring (March–April), and was typically sporadic from late spring to summer (May–July). The best fitting generalized additive mixed model (GAMM; model 2) indicated that INI significantly varied by month within each song year (July 1–June 30; *p* < 2e−16, *p* = 0.006, or *p* = 0.003; see Table 2; Fig. 2). Similar monthly trends were observed in song years one (January–June 2017), two (July 2017–June 2018), three (July 2018–June 2019), and five (July–December 2020) with no significant difference present between these years (Table 2). In contrast, the trend in song year four (July 2019–June 2020) was significantly different from the other song years (*p* = 0.0001; Table 2; Fig. 2). Therefore, trends in INI for song years one, two, three, and five will be described together while the trend for song year four will be described separately.

**Table 1:** Number of songs and INIs (in parentheses) measured per month and song year from January 2017 to December 2020 and the associated acoustic review effort.

Month	Number of songs (INIs) measured					
	*Song Year 1* *(2016–2017)*	*Song Year 2* *(2017–2018)*	*Song Year 3* *(2018–2019)*	*Song Year 4* *(2019–2020)*	*Song Year 5* *(2020–2021)*	*Totals*
July	No data	7 (550)	6 (323)	0 (0)	0 (0)	13 (873)
August	No data	9 (837)	8 (714)	6 (465)	4 (266)	27 (2282)
September	No data	7 (895)	11 (1691)	13 (852)	6 (721)	37 (4159)
October	No data	4 (578)	10 (1488)	10 (888)	9 (1308)	33 (4262)
November	No data	5 (657)	5 (642)	11 (1070)	10 (1515)	31 (3884)
December	No data	No data	10 (1240)	5 (302)	9 (1094)	24 (2636)
January	14 (1681)	No data	No data	11 (930)	No data	25 (2611)
February	12 (804)	5 (435)	2 (104)	4 (261)	No data	23 (1604)
March	9 (480)	4 (274)	7 (516)	5 (296)	No data	25 (1566)
April	4 (182)	1 (11)	3 (191)	0 (0)	No data	8 (384)
May	0 (0)	0 (0)	1 (23)	0 (0)	No data	1 (23)
June	3 (142)	0 (0)	1 (51)	0 (0)	No data	4 (193)
Totals	42 (3289)	42 (4237)	64 (6983)	65 (5064)	38 (4904)	251 (24,477)
Total days (hours) reviewed	90 (270)	145 (435)	157 (471)	169 (507)	92 (276)	653 (1959)

No Data denotes months when the buoy was not in the water and thus no archived recordings were available. Song data were not analyzed between July–December 2016 and January–June 2021.

**Table 2:** Summary of GAMMs used to assess trends in fin whale song INI.

Model # & mgcv formula	Significance of parametric coefficients					Significance of smooth terms					Model fit	
	*Term*	*Intercept*	*SE*	*t Value*	*p Value*	*Term*	*EDF*	*df*	*F statistic*	*p Value*		
1: *INI* ~ *s(Month)* + *s(SongYear)*	–	2.45	0.01	306.5	** < 2e−16**	Month	6.42	10	22.78	** < 2e−16**	*N* *R*^*2*^ *AIC*	24,477 0.42 **−**15,636
						SY	3.96	3.96	70.01	** < 2e−16**		
2: *INI* ~ *SongYear* + *s(Month* *by* = *SongYear)*	SY1	2.27	0.11	21.04	** < 2e−16**	Month By SY1	3.12	4	22.74	** < 2e−16**	*N* *R*^*2*^ *AIC*	24,477 0.52 **−**15,710
	SY2	0.14	0.11	1.23	0.22	Month By SY2	3.99	7	20.26	** < 2e−16**		
	SY3	0.13	0.11	1.18	0.24	Month by SY3	4.97	9	23.77	** < 2e−16**		
	SY4	0.41	0.11	3.80	**0.0001**	Month by SY4	4.85	7	2.99	**0.0003**		
	SY5	0.08	0.11	0.74	0.46	Month by SY5	2.20	4	2.70	**0.003**		

A total of 24,477 INIs from 251 songs were analyzed. Significant *p* values for the parametric coefficients and smooth terms are bolded. The best fitting GAMM with the highest R^2^ value and lowest AIC score was model 2. SY denotes Song Year.

**Figure 3:**
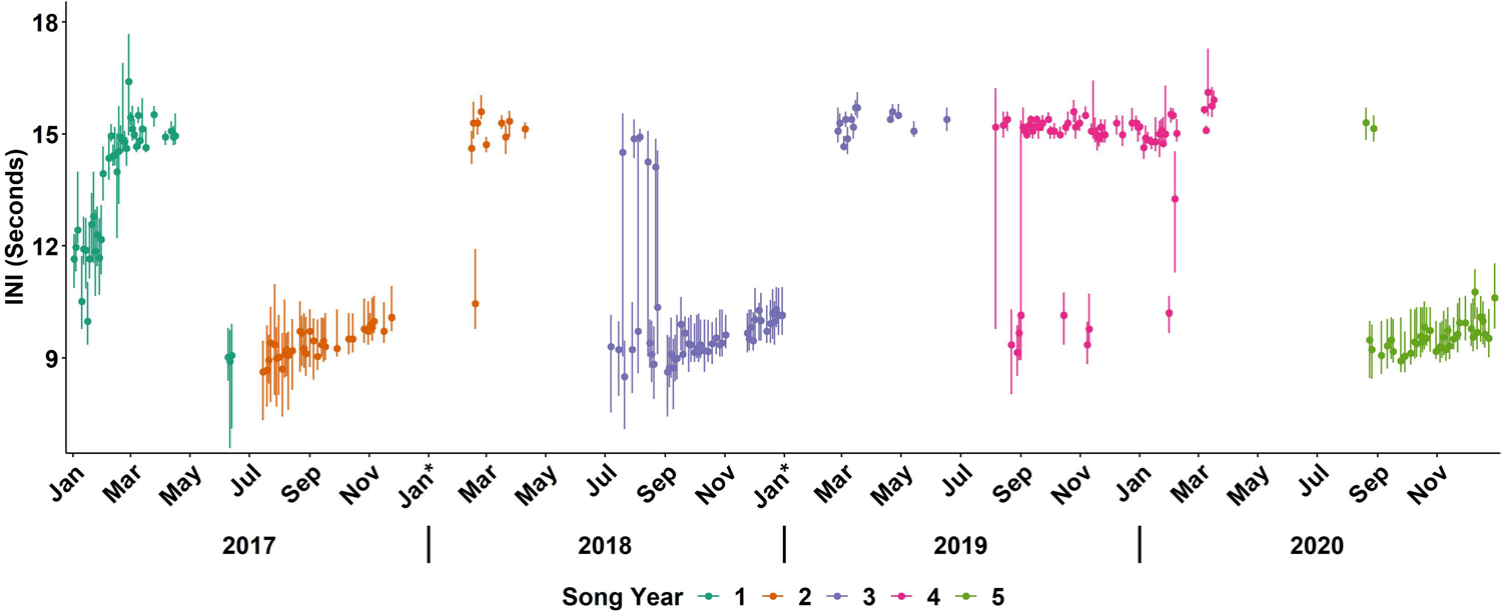
Variation in individual singer INI values by month and song year represented by the median INI with the 25th and 75th quartiles (whiskers) for January 2017–December 2020. Date was used as a proxy for distinguishing singers. Dark green represents song year one (January–June 2017), orange song year two (July 2017–June 2018), purple song year three (July 2018–June 2019), pink song year four (July 2019–June 2020), and light green song year five (July–December 2020). The asterisk on the x-axis indicates months when the buoy was not in the water during certain years, with no archival acoustic data available from December 2017, January 2018, and January 2019.

The monthly trend in INI for song years one–three and five was characterized by two stable periods with relatively constant INIs and between these were transitional periods with variable INIs (Fig. 2a–c,e). Song INIs were generally shortest from September to December (fall to winter) with INIs around 10 s, and longest from March to April (spring) with INIs around 15 s (Fig. 3; Table 3). In accordance with Morano et al.^23^, these two time periods will hereafter be referred to as the short-INI and long-INI periods, respectively. Transition periods in song INIs were observed between these two time periods, generally from January to February and/or from July to August. Furthermore, less variation in song INI, indicated by the interquartile range, was observed during the short- and long-INI periods compared to the transition periods (Fig. 3). No song data were available in May 2017, May–June 2018, and April–July 2020 due to either the absence of song or songs not meeting the minimum duration and signal to noise ratio (SNR) required to be included in analyses.

**Table 3:** Comparison of mean (± s.e.m) short- and long-INIs from January 2017 to December 2020 with those documented in 2008–2009 by Morano et al*.*^23^.

Song year	Short-INI period		Long-INI period	
	*Mean* ± *s.e.m*	*Number of Songs*	*Mean* ± *s.e.m*	*Number of songs*
1 (Jan–Jun 2017)	No data	No data	15.68 ± 0.09	13
2 (Jul 2017–Jun 2018)	9.92 ± 0.04	16	15.38 ± 0.11	5
3 (Jul 2018–Jun 2019)	9.75 ± 0.02	36	15.67 ± 0.04	11
4 (Jul 2019–Jun 2020)	14.78 ± 0.04	39	15.90 ± 0.09	5
5 (Jul–Dec 2020)	9.84 ± 0.02	34	No data	No data
2008–2009 Adapted from Morano et al*.*^23^	15.1 ± 0.11	161	9.6 ± 0.02	268

The short-INI period included INIs from songs recorded during September–December (2017–2020) or September–January^23^, while the long-INI period included INIs from songs recorded during March–May (2017–2020;^23^). Song data were not analyzed between July–December 2016 and January–June 2021.

In contrast to the other song years, distinct short- and long-INI periods were not observed in song year four (July 2019–June 2020). Instead, INIs were relatively constant throughout most of the song year with only a short period when variable INIs were observed (Figs. 2d, 3). INI patterns were more variable in August 2019 as in the other song years; however, INIs did not transition into a short-INI period. Instead, a long-INI period was observed from September 2019 to March 2020 with INIs around 15 s. Also of note, three songs (one in October and two in November) observed during this time had median INIs that were consistent with those observed during the short-INI period in the other song years (Fig. 3).

### Assessment of 20 Hz note spectral characteristics

From the 251 songs included in the INI analyses, a total of 6154 20 Hz notes measured across 100 days were classified as high SNR notes with a minimum SNR ≥ 32.3 dB (i.e., the 75th quartile SNR value across all notes; see Supplementary Table S1). The spectral characteristics of these notes did not vary by month or song year (see Supplementary Table S2). Overall, high SNR notes had a median center frequency of 19.53 Hz (range: 17.58–27.34 Hz) and a median peak frequency of 19.53 Hz (range: 15.63–27.34 Hz; see Supplementary Fig. S1). The median 90% bandwidth of the high SNR notes was 3.91 Hz (range: 1.95–11.72; see Supplementary Fig. S2).

## Discussion

Fin whale songs in the NYB during most song years (one–three and five) were characterized by seasonal shifts in INI, with songs composed of short-INIs around 10 s from September to December (i.e., the short-INI period), and long-INIs around 15 s from March to April (i.e., the long-INI period; Fig. 2a–c, e). Given that songs followed predictable seasonal shifts from short-INIs to long-INIs and had a distinct INI pattern within each season, a single song pattern was present in the NYB during these years (sensu Morano et al.^23^ and Širović et al.^24^). Furthermore, this song pattern was highly consistent with the song pattern observed almost a decade earlier in the NYB and GoM regions (see Fig. 2a–c,e, Table 3; Fig. 3 in Morano et al*.*^23^). Morano et al*.*^23^ attributed this song pattern to a single fin whale subpopulation that utilized both these regions; therefore, this song pattern will hereafter be referred to as the NYB/GoM song pattern. In contrast to song years one–three and five, the songs from song year four (July 2019–June 2020) were characterized by a single INI pattern with the majority of songs composed of long-INIs around 15 s (Fig. 3). The absence of a short-INI period during the fall and winter, as well as the consistency of the INI pattern within songs suggests that a new song pattern was present in song year four^19,24,27^. Seasonal variations in INI patterns may reflect transitions between non-reproductive and reproductive behaviors^17,18,23^, while distinct or sudden shifts in INI patterns can reflect changes in subpopulation identity^6,18,22,24^. Therefore, we hypothesize that the different song pattern observed in song year four could be due to a shift in fin whale behavior or the potential that multiple fin whale subpopulations were using the NYB.

Song patterns with seasonal shifts in INI have been observed in several locations across the northwestern Atlantic Ocean^3,6,16,23^ (see Table 4), and these seasonal shifts have been associated with changes in behavior and/or reproductive state. Fin whale reproductive activity likely occurs over winter with conception peaking in January^13,16,30^. As part of the NYB/GoM song pattern, fin whales produced songs with short-INIs leading up to and during this presumed reproductive season (see Morano et al.^23^; song years one–three and five in this study). In contrast to songs with short-INIs, long-INI patterns may correspond with non-reproductive behaviors such as foraging^17,23^. The NYB is at the southern edge of the primary feeding ground (the GoM) for fin whales in the northwest Atlantic Ocean^14,31,32^, with most observations of foraging behavior from visual survey efforts occurring in the spring and summer in the NYB^33–35^. However, in song year four the absence of a short-INI period from fall to winter could imply that fin whale behavior may not have shifted from foraging to reproduction as in the other song years. Instead, fin whales may have predominately continued to forage in the NYB, while reproductive fin whales may have utilized a different region for breeding activities. Across three years of effort, aerial surveys in the NYB only documented one occurrence of fin whales foraging during the fall^34^. This sighting occurred in November 2019 when the majority of songs were composed of long-INIs. From stranding records, it has been suggested that some calving may occur off the mid-Atlantic coast (generally south of the NYB)^31,32^. Therefore, the song year four song pattern (i.e., long-INIs throughout the year instead of seasonal shifts between short- and long-INIs) may have been a reflection of a regional shift in fin whale behavior, with fin whales primarily foraging in the NYB while reproduction occurred elsewhere.

**Table 4:** Comparison of INIs reported in the literature from studies conducted in various locations within the Atlantic Ocean.

General region or location	Recording months	Recording years	INI patterns	Source
Mid-Atlantic Ridge (MARI)	Jan, Dec	1999–2000	~ 20 s	Figure 4; Hatch and Clark^3^
Western Tropical North Atlantic (WTNA)	Jan, Feb, Mar, Dec	1993–1994	~ 12–14 s	Figure 4; Hatch and Clark^3^
Central Western North Atlantic (CWNA)	Jan, Feb	1993–1994	~ 13–14 s	Figure 4; Hatch and Clark^3^
Bermuda	All months	1958–1959; 1959–1960; 1967–1968; 1978–1979	~ 18 s (Feb–Apr); ~ 14 s (Nov–Jan)	Figure 17; Watkins et al*.*^16^
NYB/GoM	All months	2008–2009	~ 15 s (Mar–May); ~ 10 s (Sep–Jan)	Figure 3; Morano et al*.*^23^
Northwestern Atlantic (NWNA)	Jan, Feb, Mar, Apr, Nov, Dec	1994–1995; 2002–2003	~ 8–10 s; ~ 14–15 s	Figure 4; Hatch and Clark^3^
GoM	Jan, Feb, Mar, Aug, Sep, Oct, Nov, Dec	2006; 2007	~ 15 s (Jan–Mar); ~ 9 s (Sep–Jan)	Table 2 and Figure 6; Delarue et al*.*^6^
GSL	Sep, Oct, Nov, Dec	2005	~ 11–12 s (Sep–Dec)	Table 2; Delarue et al*.*^6^
Davis Strait	All months	2006–2008	~ 13.5 s (Oct–Nov)	Simon et al*.*^13^
North Eastern North Atlantic (NENA)	All months	1993–1995; 1997–2002	~ 10–12 s; ~ 18–19 s	Figure 4; Hatch and Clark^3^

The INI patterns listed represent the average INI or predominant INI patterns shown in figures and/or described in the literature. If multiple INI patterns were described and the different INI patterns were associated with specific months, those months are listed in parentheses.

The two song patterns observed during this study could potentially have been due to the presence and overlapping distribution of two fin whale subpopulations. The NYB/GoM song pattern was previously attributed to a single fin whale population that utilized both the NYB and GoM regions^23^. Additionally, the prevalence and stability of this song pattern over approximately a decade (see Fig. 2a–c, e above and Fig. 3 in Morano et al*.*^23^) suggests that it may function as a reliable indicator of the presence of fin whales from the NYB/GoM subpopulation^6,23,28^. This indicates there was a single subpopulation of fin whales, the NYB/GoM subpopulation, present in the NYB during song years one–three (January 2017–June 2019) and song year five (July–December 2020). Song year four (July 2019–June 2020) was the only year where a different song pattern was observed, and this change may indicate that a second subpopulation of fin whales was present in the NYB at this time.

The hypothesis that a second fin whale subpopulation may have been present in song year four was supported by the observation of a sudden, unexpected shift from variable to long-INIs and the brief temporal co-occurrence of two different INI patterns. At the beginning of song year four, INI patterns were more variable with a mix of songs that had median INIs around 15 s or around 10 s. This variability was similar to trends observed during the same months in other song years; however, the rapid shift from variable INI patterns to long-INI patterns at the end of August and beginning of September 2019 was unprecedented (Fig. 3). Rapid shifts in the predominant song pattern in a region may reflect changes in subpopulation presence^24^ because fin whales likely maintain their original song pattern when moving into areas where a different song was dominant (i.e., the NYB/GoM song pattern) or when multiple subpopulations are using the same habitat^6,22,24^. Prey availability is one important driver of fin whale abundance and distribution^31,32,34,36^, and shifts in prey abundance may be associated with temporary overlaps in subpopulation distribution as fin whales utilize areas with increased foraging opportunities^37^. Additionally, two distinct INI patterns were observed in songs recorded during October–November 2019. One song recorded in October and two songs recorded in November were composed of short-INIs (~ 10 s) consistent with the fall/winter INIs in the NYB/GoM song pattern (Fig. 3). All other songs recorded during these months were composed of the long-INI pattern. The co-occurrence of two INI patterns has been used to acoustically identify the presence of multiple subpopulations in other ocean basins, including the western Mediterranean Sea (i.e., the Northeast North Atlantic (NENA) and Mediterranean fin whale subpopulations)^20,22^ and across the northeast Pacific Ocean^18,24^. Thus, the brief temporal overlap between two INI patterns within the NYB in song year four could suggest that two subpopulations, the NYB/GoM subpopulation and a second unknown subpopulation, were at least temporarily present in this region. If a second subpopulation was present, it is unclear from the published literature which region this possible subpopulation may have originated from due to varying levels of recording effort across the studies, differences in recording methods, and limitations in the information provided for monthly and/or seasonal INI patterns (Table 4). Together, the rapid shift from variable to long-INIs and the brief temporal overlap of two INI patterns may provide the first acoustic evidence that at least two subpopulations of fin whales utilize habitats within the NYB, although further research is necessary to fully evaluate this hypothesis. Documenting individuals in this region, through genetics, telemetry, and/or photo-identification, as well as undertaking a more expansive comparison of fin whale song patterns across the western North Atlantic Ocean would be valuable in future efforts to examine subpopulation identity and distribution.

In addition to trends in song INI, the spectral characteristics of high SNR 20 Hz notes were evaluated because variations in center frequency, peak frequency, and/or note bandwidth have been observed spatially (i.e., between different geographic regions), temporally (i.e., across a decade), and/or between subpopulations in both the Atlantic and Pacific ocean basins^3,6,7,19,22,23,25^. For example, in the western Mediterranean Sea, INI pattern and the bandwidth of 20 Hz notes both function as strong indicators of subpopulation identity^20,22^. In the northeast Pacific Ocean, the peak frequency of 20 Hz notes decreased while song INIs increased over the span of a decade^19^. Within the NYB from January 2017 to December 2020, the center frequency, peak frequency, and 90% bandwidth of high SNR notes all remained relatively consistent with only a minimal amount of individual-level variation observed (see Supplementary Figs. S1 and S2 for further details). The lack of variation in these spectral characteristics, particularly in song year four when the song pattern was distinct from the NYB/GoM song pattern, may suggest that these measurements are not informative indicators of subpopulation identity in the northwestern Atlantic. Nonetheless, these results provide valuable baseline information on the spectral characteristics of 20 Hz notes produced by fin whales in the NYB.

Fin whale song was present year-round in the NYB from 2017 to 2020, with peaks in singing activity occurring from fall to winter and in the spring. This year-round acoustic presence of male fin whales aligns with previous research conducted both in the NYB and more broadly along the U.S. east coast^14,15,23^. Additionally, the observed peaks in singing activity coincided with specific INI patterns within the NYB/GoM song pattern, with songs composed of short-INIs during the fall to winter peak and long-INIs during the spring peak (song years one–three and five; see Morano et al*.*^23^). These seasonal changes in INI pattern likely reflect transitions between reproductive and non-reproductive behaviors, respectively^17,18,23^. In the northeast Pacific Ocean, Oleson et al*.*^18^ observed similar seasonal trends in INI patterns, and they hypothesized the higher call rate (i.e., shorter INIs) during the reproductive season could play a role in either intra- or intersexual selection. Considering that the short-INI period within the NYB/GoM song pattern led up to and overlapped with the presumed reproductive season in the northwestern Atlantic Ocean^13,16,23,30^, higher call rates or short-INIs likely have a reproductive function in this region as well. Conversely, the long-INI period and more variable INIs observed from spring to summer in the NYB/GoM song pattern may have been indicative of non-reproductive behaviors, such as foraging^17,23^. Recent visual survey efforts in this region have documented numerous occurrences of fin whale foraging behavior from spring to summer^33–35^. Collectively, these studies indicate that the NYB likely serves as an important habitat for both breeding and foraging fin whales in the northwestern Atlantic Ocean.

The NYB is a highly urbanized area facing consistent and increasing anthropogenic pressures from shipping traffic and forthcoming offshore wind energy development. Fin whales, which are listed as endangered under the U.S. Endangered Species Act, are known to be vulnerable to vessel strike^9,14,15,33,34^ and the high rate of both commercial and recreational vessel traffic in the NYB is of particular concern for this species. In conjunction with vessel traffic, noise levels in the NYB are elevated compared to other regions along the U.S. east coast^14,38^, and chronic exposure to anthropogenic noise can lead to a number of behavioral and/or physiological impacts^2,12,14,33,39^. Forthcoming offshore wind development is expected to contribute to increases in certain types of anthropogenic noise during construction activities and the risks associated with vessel traffic^29^. Wider changes in oceanographic conditions may also influence movement patterns for fin whales in the northwestern Atlantic. Davis et al*.*^15^ documented a northern shift in fin whale acoustic presence along the U.S. east coast, and this shift may be associated with changes in prey distribution driven by climatic changes. Given the scale and pace of anthropogenic activities, effective management and mitigation measures for endangered and vulnerable marine species are needed, and these measures should also consider shifts caused by changing ecosystem conditions.

Currently, only a single fin whale stock is recognized (i.e., the Western North Atlantic fin whale stock) by NOAA and the IWC for management purposes^9^. Previous PAM studies have proposed that at least two different subpopulations of fin whales are present within the northwestern Atlantic Ocean: the GSL subpopulation and the NYB/GoM subpopulation^6,23^. The NYB/GoM song pattern we observed during song years one–three and five provides further support for the hypothesis that fin whales in the NYB belong to the same subpopulation as those found within the GoM (sensu Delarue et al.^6^ and Morano et al.^23^). Thus, fin whales in the GoM and NYB should be treated as a single subpopulation for management purposes, while the GSL subpopulation should be managed separately. Managing the GSL subpopulation and the NYB/GoM subpopulation together as a single stock may leave one or both of these subpopulations vulnerable to local disturbance or extirpation^5,6^. Therefore, region specific management and mitigation practices are needed within the northwestern Atlantic Ocean to ensure that these fin whale subpopulations are adequately protected from local anthropogenic activities.

## Methods

### Study area and collection of acoustic data

The NYB encompasses waters from the coast out to the continental shelf and ranges from Cape May at the southern tip of New Jersey to Montauk at the eastern tip of Long Island, New York. Passive acoustic data were collected within the NYB using a moored buoy (Fig. 1). The buoy system contained a single, custom-built hydrophone with a flat frequency response between 8–7500 Hz, and at 2000 Hz the noise floor and hydrophone sensitivity were 36 dB re µPa/√Hz and −169.8 dB re V/µPa, respectively^40–42^. The hydrophone was operated on a duty cycle with 30 min recorded every 60 min (2017–2019) or recorded continuously (2020). Recordings were digitized and stored with a custom built digital acoustic monitoring (DMON) instrument (see Baumgartner et al*.*^41^ for further buoy system details), with a sampling rate of 2000 Hz and a 16 bit depth. Archived recordings were retrieved at the end of each deployment. The DMON and hydrophone were attached to an aluminum structure moored on the sea floor, and were consistently deployed at the same location within the NYB from January 2017 to December 2020 in a water depth of 36 m (Fig. 1).

### Song and 20 Hz note selection

A subsample of archived acoustic recordings from 2017 to 2020 were manually reviewed to assess the monthly and yearly trends in fin whale song within the NYB. Starting with the first full day after the buoy deployment, recordings from every other day were selected for review. The hours per day were also subsampled, and only six recordings (collected during hours 00:00, 04:00, 08:00, 12:00, 16:00, and 20:00) per day were reviewed. Although recordings were collected continuously in 2020, only the first 30 min from each subsampled hour were reviewed to be consistent with the data available from 2017 to 2019. Spectrograms of the subsampled recordings were visually and aurally reviewed for the presence of repeated sequences of fin whale 20 Hz notes at a frequency range from 0 to 160 Hz (Hann window, 1024 FFT, 90% overlap, with 1.95 Hz frequency and 0.05 s temporal resolutions) in Raven Pro (version 1.6.1)^43^. Repeated sequences of 20 Hz notes will hereafter be referred to as song while individual sounds will be referred to as 20 Hz notes.

During the manual review of the subsampled recordings, only fin whale songs that fit specific criteria were annotated in Raven Pro. The duty cycle of the recorder in 2017–2019 (i.e., 30 min every 60 min) made it impossible to determine if songs observed in consecutive hours were produced by the same individual, therefore only one subsampled recording with song per day was selected for annotation to ensure independence of data. If song was present in two or more subsampled recordings from a single day, the recording with the clearest, most distinct song and ideally only a single singer was selected for annotation. If multiple singers were present in the recording with the best song, only the 20 Hz notes that could be unambiguously assigned to the same song (i.e., one singer), based on the relative amplitude and general pattern in the timing of notes^3,20^, were annotated for further analyses. If there was any uncertainty in assigning the 20 Hz notes to the same singer (i.e., notes had similar amplitude or notes overlapped in time), then the song in that subsampled recording was excluded from analyses. The subsampling regime used (i.e., every other day) was longer than the typical maximum duration of an individual song sequence (up to 32 h)^6^, therefore songs recorded on different days were considered to be produced by different individuals and the date of recording was used as a proxy for singer identification. Accordingly, all songs were assigned a singer identification number based on the recording date. All annotated songs were then further evaluated based on song duration and SNR.

Only annotated songs with a minimum duration of two minutes were included in the analyses^18,24,28^. This criterion was selected because it was relatively conservative and it allowed songs with fewer 20 Hz notes but longer INIs to be retained for analyses. Although other song duration criteria have been described in the literature^6,19^, these criteria were determined to be too restrictive when considering the duty cycle of the recorder and annotated song data available for analyses in the current study.

The SNR of all annotated songs was assessed, and only songs with a mean SNR of ≥ 10 dB were included in analyses^13^. SNR was measured following a protocol developed for estimating SNR using selection tables created in Raven Pro (see^44^ for protocol details). Selections for the ‘noise’ measurements were auto-generated to be five seconds prior to and with the same frequency bounds as each 20 Hz note selection using a custom script written in RStudio (version 4.0.5)^45^. These selections were then opened in Raven Pro and the inband power (dB) measurement was calculated for each ‘noise’ and 20 Hz note selection. The conversion of inband power measurement to linear units, calculation of SNR in linear units, and conversion of SNR to decibels were then calculated using RStudio^44,45^.

To evaluate the song present in the NYB from January 2017 to December 2020, the INI between two 20 Hz notes was measured as the difference between the center time of one note and the center time of the following note^7,22,25^. Measuring INI from center time to center time of sequential 20 Hz notes was selected to avoid the influence of variable SNR within and between song sequences^25^. During the initial evaluation of song INI, all INIs < 4.5 s and > 30 s were removed to exclude long intervals that were potentially due to the whale surfacing to breathe, missed notes that overlapped with other sounds, and/or multipath propagation of notes^19,25^.

The spectral characteristics of annotated 20 Hz notes were assessed using center frequency (Hz), peak frequency (Hz), and 90% bandwidth (Hz). These note measurements were selected because variations in these measurements have been observed between different geographic regions and/or subpopulations^6,7,22,23,25^. Furthermore, center frequency, peak frequency, and 90% bandwidth are robust measurements (i.e., measurements that are not as influenced by the exact bounds of the selection) in Raven Pro^46^. To begin, the SNR measurements across all annotated songs included in the INI analyses were pooled and the overall median, 25th quartile, and 75th quartile values for SNR were calculated. Since variable SNR and sound propagation would bias the note measurements^27,39^, only 20 Hz notes where the SNR was greater than or equal to the 75th quartile value (hereafter referred to as high SNR notes) and only days with a minimum of 10 measured high SNR notes were included in the spectral characteristic analyses.

### Analysis of monthly and yearly trends in INI

To evaluate monthly and yearly variability in fin whale song INI, generalized additive mixed models (GAMMs)^47–50^ were fitted to the data using the *gamm* function in the *mgcv* package^49,50^ in RStudio. The response variable was INI and the two predictor variables were song year and month the song was recorded. Song was most prevalent from fall to spring, thus song year was defined by the singing season^18^. Each song year began on July 1 and ended on June 30. A gamma distribution with log link function was used to model the data because this family is appropriate for interval data, such as INI, and provides relative flexibility. To account for the repeated measurements of INI for each singer, the identification number of singers (assigned based on the date the song was recorded) was set as a random effect and used to define the correlation structure within the models. Year was fit as a spline with an upper limit of five degrees of freedom and month as a cyclic cubic spline with an upper limit of 12 degrees of freedom to match the number of unique values for each predictor. Models were generated to evaluate trends in INI by (1) month and song year, and (2) month by song year^48,51^. The final model was selected based on minimum AIC value. The fit of this model was assessed using Q-Q plots and plots of the residuals.

To further examine trends in INI pattern, the daily variation in INI (i.e., the INI pattern for each song) by month and song year was examined by calculating and then visualizing the median, 25th quartile, and 75th quartile INI values per day (i.e., per song) in RStudio, where larger interquartile ranges (IQR) indicate that more variation in INI pattern was present.

### Analysis of 20 Hz note spectral characteristics

An initial examination of the center frequency, peak frequency, and bandwidth of high SNR notes indicated that some non-linear trends may be present within the data; therefore, the monthly and yearly trends in these spectral characteristics were evaluated using generalized additive models (GAMs). GAMs were fitted to the data using the *gam* function in the *mgcv* package^49,50^. A separate model was fit to the data for each spectral characteristic, thus the response variable in each model was either center frequency, peak frequency, or bandwidth. The spectral characteristics were all evaluated using song year and month as the predictor variables. Song year was fit as a spline with an upper limit of five degrees of freedom to match the number of unique values available. None of the 20 Hz notes recorded in May and June had an adequate SNR to be included in analyses, thus month was fit as a cyclic cubic spline with an upper limit of ten degrees of freedom. As the data were continuous with a skewed distribution, the models were run using a Gamma distribution with an identity link function. To account for repeated measurements within a day, the date the notes were recorded was set as the random effect. The fit of all models was evaluated using Q-Q plots and residual plots.

### Supplementary Information

Supplementary Information.

## Data Availability

The datasets generated and analyzed during the current study are available from the corresponding author on reasonable request.

## References

[1] Halpern BS (2008). A global map of human impact on marine ecosystems. Science.

[2] Hawkins ER (2017). Best practice framework and principles for monitoring the effect of coastal development on marine mammals. Front. Mar. Sci..

[3] Hatch, L. T. & Clark, C. W. Acoustic differentiation between fin whales in both the North Atlantic and North Pacific Oceans, and integration with genetic estimates of divergence. In *Paper SC/56/SD8 presented to IWC Scientific Committee, June 2004*. 37 pp. Available from publications@iwc.int (2004).

[4] McDonald MA, Mesnick SL, Hildebrand JA (2006). Biogeographic characterization of blue whale song worldwide: Using song to identify populations. J. Cetacean Res. Manag..

[5] Clapham PJ, Aguilar A, Hatch LT (2008). Determining spatial and temporal scales for management: Lessons from whaling. Mar. Mammal Sci..

[6] Delarue J, Todd SK, Van Parijs SM, Iorio LD (2009). Geographic variation in Northwest Atlantic fin whale (*Balaenoptera physalus*) song: Implications for stock structure assessment. J. Acoust. Soc. Am..

[7] Rankin, S. *et al.* Methods for characterizing fin whale song notes for comparative studies of geographic variation in song. In *NOAA Technical Memorandum NMFS-SWFSC-592,* 33 pp. (2018).

[8] Watkins WA (1981). Activities and underwater sounds of fin whales. Sci. Rep. Whales Res. Inst..

[9] NOAA (National Oceanographic and Atmospheric Administration). *Fin whale (Balaenoptera physalus): Western North Atlantic Stock*. https://media.fisheries.noaa.gov/2022-08/Fin%20Whale-West%20N%20Atl%20Stock_SAR%202021.pdf (2022).

[10] Curtis KR, Howe BM, Mercer JA (1999). Low-frequency ambient sound in the North Pacific: Long time series observations. J. Acoust. Soc. Am..

[11] Nieukirk SL, Stafford KM, Mellinger DK, Dziak RP, Fox CG (2004). Low-frequency whale and seismic airgun sounds recorded in the mid-Atlantic Ocean. J. Acoust. Soc. Am..

[12] Nieukirk SL (2012). Sounds from airguns and fin whales recorded in the mid-Atlantic Ocean, 1999–2009. J. Acoust. Soc. Am..

[13] Simon M, Stafford KM, Beedholm K, Lee CM, Madsen PT (2010). Singing behavior of fin whales in the Davis Strait with implications for mating, migration and foraging. J. Acoust. Soc. Am..

[14] Muirhead CA (2018). Seasonal acoustic occurrence of blue, fin, and North Atlantic right whales in the New York Bight. Aquat. Conserv. Mar. Freshw. Ecosyst..

[15] Davis GE (2020). Exploring movement patterns and changing distributions of baleen whales in the western North Atlantic using a decade of passive acoustic data. Glob. Change Biol..

[16] Watkins WA, Tyack P, Moore KE, Bird JE (1987). The 20-Hz signals of finback whales (*Balaenoptera physalus*). J. Acoust. Soc. Am..

[17] Croll DA (2002). Only male fin whales sing loud songs. Nature.

[18] Oleson EM, Širović A, Bayless AR, Hildebrand JA (2014). Synchronous seasonal changes in fin whale song in the North Pacific. PLoS ONE.

[19] Weirathmueller MJ (2017). Spatial and temporal trends in fin whale vocalizations recorded in the NE Pacific Ocean between 2003–2013. PLoS ONE.

[20] Pereira A, Harris D, Tyack P, Matias L (2020). Fin whale acoustic presence and song characteristics in the seas to the southwest of Portugal. J. Acoust. Soc. Am..

[21] Thompson PO, Findley LT, Vidal O (1992). 20-Hz pulses and other vocalizations of fin whales, *Balaenoptera physalus*, in the Gulf of California, Mexico. J. Acoust. Soc. Am..

[22] Castellote M, Clark CW, Lammers MO (2012). Fin whale (*Balaenoptera physalus*) population identity in the Western Mediterranean Sea. Mar. Mammal Sci..

[23] Morano JL (2012). Seasonal and geographical patterns of fin whale song in the western North Atlantic Ocean. J. Acoust. Soc. Am..

[24] Širović A, Oleson EM, Buccowich J, Rice A, Bayless AR (2017). Fin whale song variability in southern California and the Gulf of California. Sci. Rep..

[25] Archer FI, Rankin S, Stafford KM, Castellote M, Delarue J (2020). Quantifying spatial and temporal variation of North Pacific fin whale (*Balaenoptera physalus*) acoustic behavior. Mar. Mammal Sci..

[26] Watkins WA (2000). Seasonality and distribution of whale calls in the North Pacific. Oceanography.

[27] Helble TA (2020). Fin whale song patterns shift over time in the central North Pacific. Front. Mar. Sci..

[28] Wood M, Širović A (2022). Characterization of fin whale song off the Western Antarctic Peninsula. PLoS ONE.

[29] Kraus, S. D., Kenney, R. D. & Thomas, L. A framework for studying the effects of offshore wind development on marine mammals and turtles. In *Report for the Massachusetts Clean Energy Center and the Bureau of Ocean Energy Management.* https://www.boem.gov/sites/default/files/environmental-stewardship/Environmental-Studies/Renewable-Energy/A-Framework-for-Studying-the-Effects.pdf (2019).

[30] Lockyer C (1984). Review of baleen whale (Mysticeti) reproduction and implications for management. Rep. Int. Whal. Comm..

[31] Hain JHW, Ratnaswamy MJ, Kenney RD, Winn HE (1992). The fin whale, *Balaenoptera physalus*, in waters of the Northeastern United States continental shelf. Rep. Int. Whal. Comm..

[32] LaBrecque E, Curtice C, Harrison J, Van Parijs SM, Halpin PN (2015). Biologically important areas for cetaceans within U.S. waters—East Coast region. Aquat. Mamm..

[33] King CD, Chou E, Rekdahl ML, Trabue SG, Rosenbaum HC (2021). Baleen whale distribution, behaviour and overlap with anthropogenic activity in coastal regions of the New York Bight. Mar. Biol. Res..

[34] Lomac-MacNair KS, Zoidis AM, Ireland DS, Rickard ME, McKown KA (2022). Fin, humpback, and minke whale foraging events in the New York Bight as observed from aerial surveys, 2017–2020. Aquat. Mamm..

[35] Zoidis AM (2021). Distribution and density of six large whale species in the New York Bight from monthly aerial surveys 2017 to 2020. Cont. Shelf Res..

[36] Payne PM (1990). Recent fluctuations in the abundance of baleen whales in the southern Gulf of Main in relation to changes in selected prey. Fish. Bull..

[37] Coakes A (2005). Photographic identification of fin whales (*Balaenoptera physalus*) off the Atlantic coast of Nova Scotia, Canada. Mar. Mammal Sci..

[38] Rice AN (2014). Variation of ocean acoustic environments along the western North Atlantic coast: A case study in context of the right whale migration route. Ecol. Inform..

[39] Castellote M, Clark CW, Lammers MO (2012). Acoustic and behavioral changes by fin whales (*Balaenoptera physalus*) in response to shipping and airgun noise. Biol. Conserv..

[40] Baumgartner MF (2013). Real-time reporting of baleen whale passive acoustic detections from ocean gliders. J. Acoust. Soc. Am..

[41] Baumgartner MF (2019). Persistent near real-time passive acoustic monitoring for baleen whales from a moored buoy: System description and evaluation. Methods Ecol. Evol..

[42] Johnson, M. & Hurst, T. The DMON: An open-hardware/open-software passive acoustic detector. In *3rd International Workshop on the Detection and Classification of Marine Mammals using Passive Acoustics, Boston, Massachusetts, USA* (2007).

[43] K. Lisa Yang Center for Conservation Bioacoustics. *Raven Pro: Interactive Sound Analysis Software (version 1.6.1)*. https://ravensoundsoftware.com/ (2023).

[44] K. Lisa Yang Center for Conservation Bioacoustics. *Signal-to-noise ratio (SNR)—User Protocol*. https://ravensoundsoftware.com/knowledge-base/signal-to-noise-ratio-snr/ (2023).

[45] RStudio Team. *RStudio: Integrated Development for R (version 4.0.5)*. http://www.rstudio.com/ (2020).

[46] Charif, R. A., Waack, A. M. & Strickman, L. M. *Raven Pro 1.4 User’s Manual*. https://ravensoundsoftware.com/knowledge-base/comprehensive-raven-pro-1-4-users-manual/ (2010).

[47] Shadish WR, Zuur AF, Sullivan KJ (2014). Using generalized additive (mixed) models to analyze single case designs. J. Sch. Psychol..

[48] Pedersen EJ, Miller DL, Simpson GL, Ross N (2019). Hierarchical generalized additive models in ecology: An introduction with mgcv. PeerJ.

[49] Wood S (2017). Generalized Additive Models: An introduction with R.

[50] Wood, S. *mgcv: Mixed GAM computation vehicle with automatic smoothness estimation*. https://cran.r-project.org/web/packages/mgcv/mgcv.pdf (2021).

[51] Ahonen H (2021). Interannual variability in acoustic detection of blue and fin whale calls in the Northeast Atlantic High Arctic between 2008 and 2018. Endanger. Species Res..

