# Supplementary material for: Fin whale song characteristics and potential subpopulation identity in the New York Bight
Source: Sci Rep. 2024 Feb 13;14:2931. doi: 10.1038/s41598-024-52228-8 (PMC10864287; doi:10.1038/s41598-024-52228-8)
Supplement: Supplementary file 1 — Supplementary Information. [file 41598_2024_52228_MOESM1_ESM.pdf]

Fin whale song characteristics and potential subpopulation identity in the New York Bight

Carissa D. King-Nolan*, Melinda L. Rekdahl, Anita Murray, Samantha Strindberg, Mark F. Baumgartner, Howard C. Rosenbaum

Supplementary Information

Supplementary Table S1. The number of days and high SNR notes (in parentheses) measured per month and song year from January 2017 – December 2020. No Data denotes months when the buoy was not in the water and thus no archived recordings were available. Song data were not analyzed between July – December 2016 and January – June 2021.

	**Number of Days (High SNR Notes) Measured**					
**Month**	*Song Year 1 (2016–2017)*	*Song Year 2 (2017–2018)*	*Song Year 3 (2018–2019)*	*Song Year 4 (2019–2020)*	*Song Year 5 (2020–2021)*	***Totals***
July	No Data	2 (76)	1 (46)	0 (0)	0 (0)	**3 (122)**
August	No Data	4 (184)	5 (275)	0 (0)	0 (0)	**9 (459)**
September	No Data	2 (169)	9 (794)	1 (99)	0 (0)	**12 (1,062)**
October	No Data	2 (274)	5 (486)	4 (218)	3 (238)	**14 (1,216)**
November	No Data	1 (112)	2 (147)	4 (91)	3 (79)	**10 (429)**
December	No Data	No Data	6 (426)	0 (0)	6 (525)	**12 (951)**
January	10 (578)	No Data	No Data	6 (379)	No Data	**16 (957)**
February	10 (343)	3 (56)	0 (0)	0 (0)	No Data	**13 (399)**
March	4 (178)	1 (99)	2 (61)	2 (101)	No Data	**9 (439)**
April	1 (44)	0 (0)	1 (76)	0 (0)	No Data	**2 (120)**
May	0 (0)	0 (0)	0 (0)	0 (0)	No Data	**0 (0)**
June	0 (0)	0 (0)	0 (0)	0 (0)	No Data	**0 (0)**
**Totals**	**25 (1,143)**	**15 (970)**	**31 (2,311)**	**17 (888)**	**12 (842)**	**100 (6,154)**

Supplementary Table S2. Summary of GAMs used to assess monthly and yearly trends in the spectral characteristics of high SNR notes. A total of 6,154 notes measured across 100 days were analyzed. Significant p-values for the parametric coefficients and smooth terms are bolded. The response variable in each model was center frequency (CF), peak frequency (PF), or bandwidth (BW).

**Model**	**Significance of parametric coefficients**				**Significance of Smooth Terms**					**Model Fit**		
	Intercept	*SE*	*t - value*	*p - value*	*Term*	*EDF*	*df*	*F statistic*	*p - value*	*N*	R^2	*Deviance Explained*
CF ~ s(Month) + s(Song Year)	19.80	0.06	306.8	**<2e-16**	Song Year	1	1	1.36	0.24	6,154	0.57	59.9%
					Month	0.002	8	0.003	0.33			
PF ~ s(Month) + s(Song Year)	19.82	0.07	289.5	**<2e-16**	Song Year	1	1	1.65	0.20	6,154	0.56	59%
					Month	4.09 e-4	8	0.00	0.38			
BW ~ s(Month) + s(Song Year)	4.45	0.08	53.06	**<2e-16**	Song Year	1	1	2.22	0.14	6,154	0.44	50.3%
					Month	0.48	8	64.30	0.46			

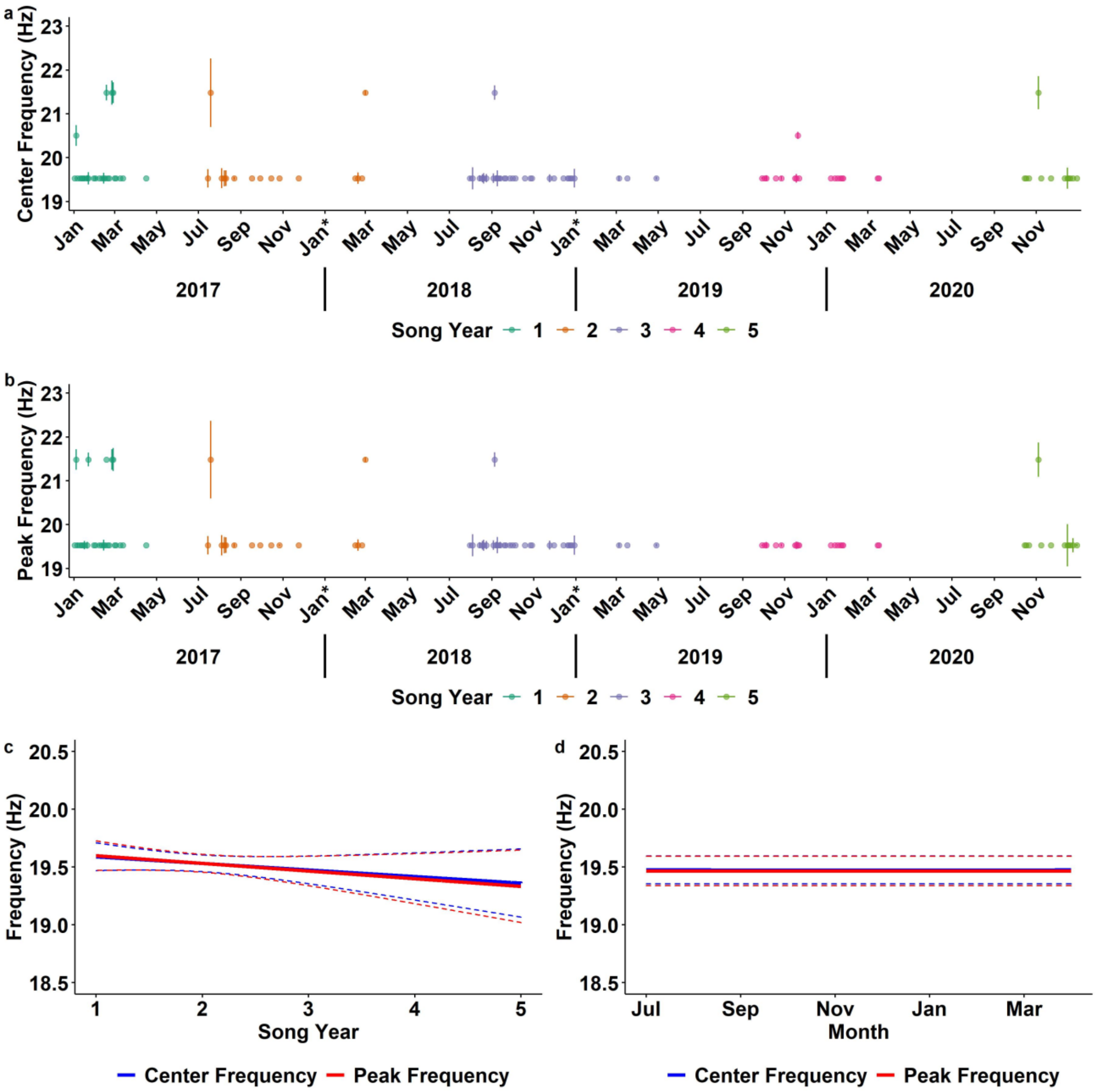

Supplementary Figure S1. Observed and predicted variation in center frequency (Hz) and peak frequency (Hz) from January 2017 – December 2020. Median (± s.e.m) values for (a) center frequency and (b) peak frequency by date of recording. Dark green represents song year one (January – June 2017), orange song year two (July 2017 – June 2018), purple song year three (July 2018 – June 2019), pink song year four (July 2019 – June 2020), and light green song year five (July – December 2020). The solid line shows predicted trends from the GAMs for center and peak frequency by (c) song year and (d) month with the dotted lines indicating the 95% confidence interval of the model. Predictions for center frequency are in blue and peak frequency in red. No significant difference by song year or month were indicated by the GAMs for center and peak frequency. The asterisk on the x-axis indicates months when the buoy was not in the water, with no archival acoustic data available from December 2017, January 2018, and January 2019.

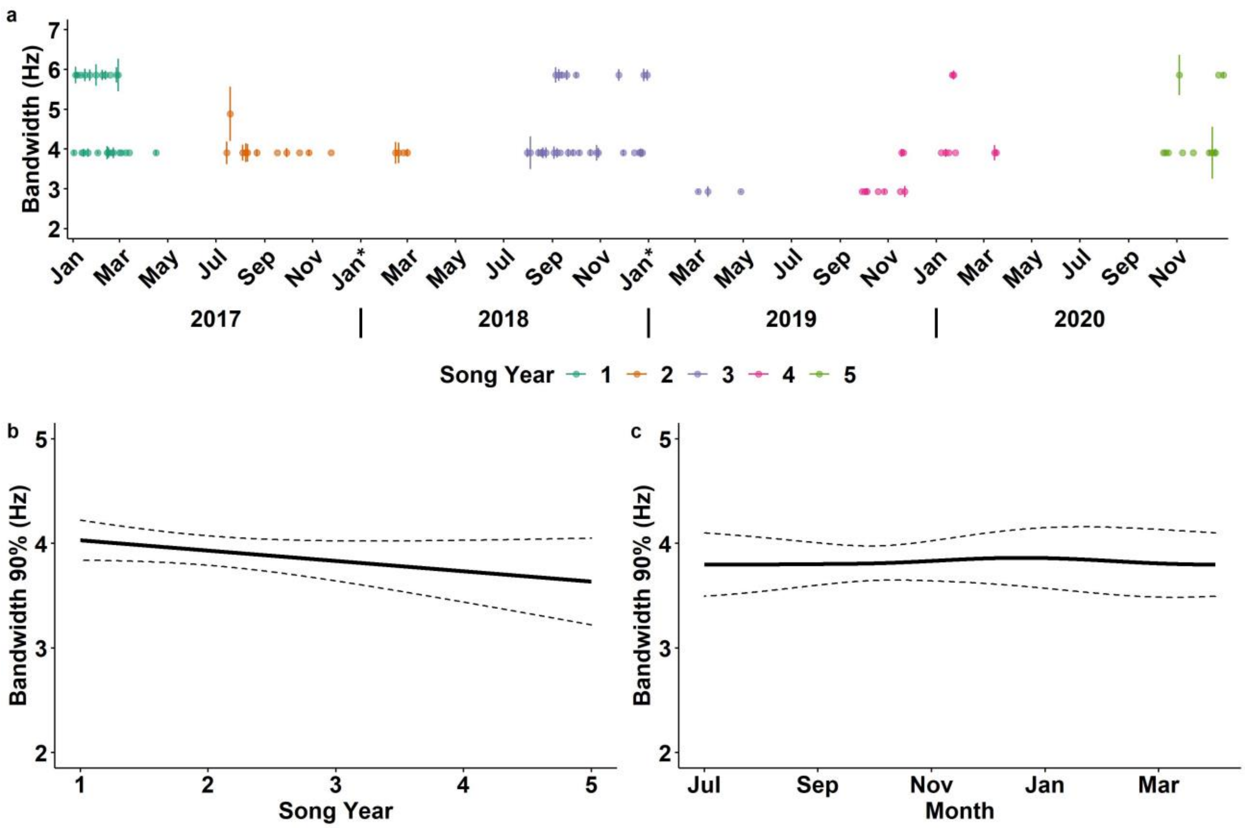

Supplementary Figure S2. Observed and predicted variation in bandwidth (Hz) from January 2017 – December 2020. Median (± s.e.m) values for (a) bandwidth are shown by date of recording. Dark green represents song year one (January – June 2017), orange song year two (July 2017 – June 2018), purple song year three (July 2018 – June 2019), pink song year four (July 2019 – June 2020), and light green song year five (July – December 2020). The solid line shows predicted trends from the GAM for bandwidth by (b) song year and (c) month with the dotted lines indicating the 95% confidence interval of the model. No significant difference by song year or month was indicated by the GAM for note bandwidth. The asterisk on the x-axis indicates months when the buoy was not in the water, with no archival acoustic data available from December 2017, January 2018, and January 2019.
